# Osteocalcin Alleviates Lipopolysaccharide-Induced Acute Inflammation via Activation of GPR37 in Macrophages

**DOI:** 10.3390/biomedicines10051006

**Published:** 2022-04-27

**Authors:** Zhengjiang Qian, Chunhua Liu, Hongchao Li, Haiyang Yang, Jianhao Wu, Jing Liu, Yanjiao Li, Xuhui Chen, Jianyang Xu, Xiang Li

**Affiliations:** 1 Guangdong Provincial Key Laboratory of Brain Connectome and Behavior, CAS Key Laboratory of Brain Connectome and Manipulation, Shenzhen Key Laboratory of Viral Vectors for Biomedicine, the Brain Cognition and Brain Disease Institute, Shenzhen Institute of Advanced Technology, Chinese Academy of Sciences, Shenzhen-Hong Kong Institute of Brain Science-Shenzhen Fundamental Research Institutions, Shenzhen 518055, China; zj.qian@siat.ac.cn (Z.Q.); ch.liu@siat.ac.cn (C.L.); hc.li1@siat.ac.cn (H.L.); hy.yang1@siat.ac.cn (H.Y.); 2 University of Chinese Academy of Sciences, Beijing 100049, China; 3 Department of Traditional Chinese Medicine, Shenzhen University General Hospital, Shenzhen 518055, China; wujianhao@szu.edu.cn (J.W.); liujing2@szu.edu.cn (J.L.); xujianyang@szu.edu.cn (J.X.); 4 Faculty of Life and Health Sciences, Shenzhen Institute of Advanced Technology, Chinese Academy of Sciences, Shenzhen 518055, China; yj.li@siat.ac.cn; 5 Department of Neurology, Peking University Shenzhen Hospital, Shenzhen 518000, China; 1589@pkuszh.com

**Keywords:** osteocalcin, GPR37, macrophage, lipopolysaccharide, acute inflammation

## Abstract

The G protein-coupled receptor 37 (GPR37) has been reported to be expressed in macrophages and the activation of GPR37 by its ligand/agonist, and it can regulate macrophage-associated functions and inflammatory responses. Since our previous work identified that osteocalcin (OCN) acts as an endogenous ligand for GPR37 and can elicit various intracellular signals by interacting with GPR37, we thus hypothesized that OCN may also play a functional role in macrophage through the activation of GPR37. To verify the hypothesis, we conducted a series of in vivo and in vitro studies in lipopolysaccharide (LPS)-challenged mice and primary cultured macrophages. Our results reveal that the *OCN* gene deletion (*OCN*^−/−^) and wild type (WT) mice showed comparable death rates and inflammatory cytokines productions in response to a lethal dose of LPS exposure. However, the detrimental effects caused by LPS were significantly ameliorated by exogenous OCN treatments in both WT and *OCN*^−/−^ mice. Notably, the protective effects of OCN were absent in *GPR37*^−/−^ mice. In coordination with the in vivo results, our in vitro studies further illustrated that OCN triggered intracellular responses via GPR37 in peritoneal macrophages by regulating the release of inflammatory factors and macrophage phagocytic function. Finally, we exhibited that the adoptive transfer of OCN-treated macrophages from WT mice significantly inhibits the release of pro-inflammatory cytokines in *GPR37*^−/−^ mice exposed to LPS. Taken together, these findings suggest a protective role of OCN against LPS-caused acute inflammation, by the activation of GPR37 in macrophages, and provide a potential application of the activation of the OCN/GPR37 regulatory axis as a therapeutic strategy for inflammatory diseases.

## 1. Introduction

Osteocalcin (OCN), also known as bone γ-carboxyglutamic acid (Gla) protein or BGP, is the most abundant, non-collagenous protein indigenous to the mineralized matrix of bone and dentin [1]. This protein, containing 46–50 amino acids (depending on the species), with three highly conserved glutamyl residues (Glu) in its mature form, is predominantly expressed and produced by osteoblasts in vertebrates [2,3]. Recent accumulating evidences indicate that OCN can act as a multifunctional hormone and regulate various kinds of physiological processes and biological activities [3,4,5,6,7]. In peripheral organs, OCN has been showed to play critical roles in modulation of lipid metabolism [8,9], insulin and glucose homeostasis [10,11,12], energy expenditure [13,14,15], vascular diseases [16,17,18], male fertility [19,20], diabetes pathogenesis [21], and exercise adaptation [22,23]. Moreover, OCN is also proved to be essential for the neuronal development and oligodendrocyte myelination in the central nervous system [24,25,26,27], thus regulating hippocampal memory and cognition, and motor behavior [6,28,29].

Interestingly, there are further controversial studies arguing for a regulatory role of OCN in inflammatory responses. On one hand, OCN is found to be inversely related to pro-inflammatory cytokines, displaying an anti-inflammatory role in the regulation of inflammation. This is evidenced by several clinical studies showing that serum OCN level was negatively correlated with systemic inflammatory markers, such as interleukin-6 (IL-6) and C-reactive protein (CRP), in patients with diabetes, obesity, or metabolic syndrome [30,31,32,33,34,35,36]. Moreover, infusion of OCN could substantially downregulate inflammatory related genes (e.g., tumor necrosis factor α (TNFα), IL-1β, and IL-6) and transcription factors in a monosodium glutamate-induced mouse model of obesity [37]. Similarly, the anti-inflammatory effect of OCN was also observed in primary cultured adipocytes stimulated with TNFα [37,38]. On the other hand, a few studies argued that OCN has no effect or even positively associated with inflammation [39,40,41]. For example, a recent study showed that OCN has no functional effects on human vascular cells (i.e., aortic endothelial cells and smooth muscle cells) with acute or chronic inflammation, as induced by interferon-γ and TNFα [40]. In atherosclerotic plaques of coronary arteries, OCN level increased significantly during calcification of atherosclerotic plaques, which is characterized by activation of inflammatory processes and release of pro-inflammatory cytokines [39]. Up to now, these conflicting results still remain and keep emerging, which complicate the understanding of OCN’s function and potential application in inflammation.

The G protein-coupled receptor 37 (GPR37) is originally recognized as a parkin-associated endothelin-like receptor (Pael-R) because it is a substrate of the E3 ubiquitin ligase parkin [42,43]. The receptor is extensively expressed in different regions of the brain and has been implicated in certain neurological disorders such as Parkinson’s disease [44,45,46,47], autism [48,49], depression, and bipolar disorder [50,51,52]. Moreover, it has been widely studied that GPR37 also plays pivotal roles in regulating physiological functions of neurons and glial cells, such as dopaminergic signaling [53,54] and oligodendrocyte myelination [55,56]. So far, several peptides have been identified as the ligands for GPR37, including head activator (HA) [57], prosaposin (PSAP) [58], and regenerating islet-derived family member 4 (REG4) [59]. These proposed ligands can trigger intracellular down-stream signal transduction through an interaction with GPR37 and, thus, regulate many biological functions [58,60]. It has been reported that GPR37 is coupled with Gi/o signaling pathway, and the activation of GPR37 by its ligands can trigger cellular downstream signal transduction through the regulation of pERK level, cAMP release, and calcium (Ca^2+^) influx [24,55,57,58].

Recent studies showed that GPR37 is expressed in macrophages and plays a protective role via the regulation of inflammation in sepsis, bacterial infection, inflammatory pain, and ischemic stroke [61,62,63]. The activation of GPR37, by its ligand neuroprotectin D1 (NPD1) or agonist artesunate (ARU), can substantially reduce these inflammation-related phenotypes [61,62]. Coincidently, our previous work identified that OCN is an endogenous ligand for GPR37 that elicits intracellular responses in the central nervous system [24]. Therefore, we hypothesized that OCN may also confer a functional role via GPR37 in macrophages.

In the present work, we showed that OCN treatment significantly alleviated lipopolysaccharide (LPS)-induced acute inflammation in WT and *OCN*^−/−^ mice, while the protective action of OCN was absent in *GPR37*^−/−^ mice. Moreover, the in vitro studies further illustrated that OCN triggers intracellular responses via interactions with GPR37 in peritoneal macrophages, thus regulating inflammatory factors release and phagocytic functions. Finally, the adoptive transfer of OCN-treated macrophages from WT mice could significantly suppress the production of pro-inflammatory cytokines in LPS-stimulated *GPR37*^−/−^ mice. Overall, our results demonstrated that OCN plays an anti-inflammatory role in LPS-induced acute inflammation through the activation of GPR37, providing a potential strategy for the treatment of acute inflammatory conditions via the OCN/GPR37 regulatory axis.

## 2. Materials and Methods

### 2.1. Animals and Treatments

All experimental procedures were performed according to protocols approved by the Institutional Animal Care and Use Committee (SIAT-IACUC-20190715-NS-NTPZX-QZJ-A0603) at Shenzhen Institute of Advanced Technology (SIAT), Chinese Academy of Science (CAS). The source of *OCN*^−/−^ and *GPR37*^−/−^ mice have been described in our previous study [24]. All the mice were bred in specified-pathogen-free facilities at SIAT with 12-h dark/12-h light cycle, with free access to food, and water *ad libitum*. Adult male and female mice (8–10 weeks) were used to produce LPS models and to isolate peritoneal macrophages for in vitro studies.

### 2.2. Serum Inflammatory Cytokines Analysis

By the end of the experiment, mice were executed, and peripheral blood was collected from eyes. The serum levels of inflammatory cytokines, including TNFα and IL-6, were analyzed by their specific ELISA kits (4A biotech, Beijing, China), according to the manufacturers’ instructions.

### 2.3. Flow Cytometry

Peripheral blood was collected from the retroorbital vein after isoflurane anesthesia by using microcentrifuge tubes containing heparin sodium as anti-coagulants. Spleen and lymph nodes were harvested and gently pressed through a 70-µm nylon mesh cell strainer (Fisher) with a sterile plastic plunger (BD Biosciences) to yield a single cell suspension. Mononucleated cells from spleen, lymph, and hemolyzed peripheral blood were treated with Red Blood Cell Lysis Buffer (eBioscience, San Diego, CA, USA) and stained with antibodies. Additionally, 4′, 6-diamidino-2-phenylindole (DAPI) was used for the assessment of cell viability. Phenotypic analysis was performed with the following fluorochrome-conjugated anti-mouse antibodies: anti-CD11c APC (clone N418, catalog no. 117310), anti-F4/80 Alexa Fluor^®^ 488 (clone BM8, catalog no. 123120) from Biolegend (San Diego, CA, USA); anti-CD45 APC-Cy7 (clone 30-F11, catalog no. 557659), anti-CD11b PE-CF594 (clone M1/70, catalog no. 562287), anti-Ly6G PE (clone 1A8, catalog no. 551461), from BD Biosciences (San Jose, CA, USA). Analysis was performed using CytExpert software on a CytoFLEX flow cytometer (Beckman Coulter, Brea, CA, USA). Flow cytometry data were analyzed with Flowjo software (FlowJo, LLC, Ashland, OR, USA). The dendritic cells, neutrophils, and macrophages were identified as CD11b^+^CD11c^+^CD45^+^, CD11b^+^Ly6G^+^CD45^+^ and CD11b^+^F4/80^+^CD45^+^, respectively.

### 2.4. Peptides

Mouse osteocalcin (OCN) peptides were synthesized in the Beijing Genomics Institute (BGI, Beijing, China). The complete amino acid sequence is as follows: YLGASVPSPDPLEPTREQCELNPACDELSDQYGLKTAYKRIYGITI. Prosaptide TX14(A) was purchased from Sigma-Aldrich (Cat# SML1417). All the peptides have showed biological activity, as described in previous work [24].

### 2.5. Cell Culture and Treatment

The mouse macrophage cell line RAW264.7 were purchased from the American Type Culture Collection (ATCC, Manassas, VA). Cells were cultured in Dulbecco’s modified Eagle’s medium (DMEM), containing 10% FBS and 1% penicillin-streptomycin in humidified atmosphere with 5% CO_2_ at 37 °C. The peritoneal macrophage cells isolation and culture were performed as described previously [64,65]. In brief, mice were injected intraperitoneally with 4 mL of 4% thioglycollate broth from Sigma-Aldrich (Cat# 70157). After 3 days of inoculation, the peritoneal macrophages were harvested with cold PBS from the peritoneal cavity. Peritoneal macrophages were cultured in RPMI 1640 medium supplemented 10% FBS. By adhering for 2 h, the adherent macrophages were used for OCN treatment and subsequent experimental analysis. 

### 2.6. Adoptive Transfer of Macrophage

The adoptive transfer experiment was conducted as previously described [61]. Briefly, the cultured peritoneal macrophages from WT and GPR37^−/−^ mice were treated with OCN (10 nM) for 24 h, and then, they were harvested with PBS, which was intraperitoneally injected into GPR37^−/−^ at 2 × 10^5^ cells.

### 2.7. RNA Extraction and qRT-PCR

The RNA extraction and qRT-PCR were carried out, as described, in previous studies [24,64]. Briefly, extraction of total RNA was performed according to the manufacturer’s instructions by TRIeasy™ LS Total RNA Extraction Reagent (YEASEN, Shanghai, China). RNA concentration was determined using a NanoDrop 2000c Spectrophotometer (Thermo Fisher Scientific, Rockford, IL, USA). Then, 2 μg of total RNA were used for the synthesis of the first strand cDNA and the real-time quantitative PCR experiments were performed on a QuantStudio PCR system (Applied Biosystems, Foster City, CA, USA) using the Hieff Unicon Universal blue SYBR Green Master mix (YEASEN, Shanghai, China) with specific gene primers (Table 1). The expression of GAPDH was taken as an internal normalization control, and its stability among different samples is subjected to MIQE guidelines [66]. Three independent experiments were performed, with triplicate reactions of each sample, and the results were obtained using the 2^−^^△△^^Ct^ method. 

### 2.8. Western Blotting

The immunoblotting was carried out, as described, in our previous work [24,64]. Protein samples were prepared using RIPA lysis buffer appended with a cocktail of protease inhibitor (Roche, Basel, Switzerland). The protein concertation was determined using a BCA protein assay kit (Beyotime, Shanghai, China). Additionally, 20 µg of the total protein sample were loaded for 10% sodium dodecyl sulphate-polyacrylamide gel electrophoresis (SDS-PAGE), which were subjected to a PVDF membrane transfer. The membrane was blocked with 5% BSA for 1 h at room temperature, followed by incubation with a specific primary antibody at 4 °C overnight. The primary antibodies included anti-NF-κB-p65 (Cat#ab16502, Abcam, Cambridge, UK), anti-NF-κB-p-p65 (S536) (Cat# ab76302, Abcam, Cambridge, UK), anti-tERK (Cat#8544, CST, Boston, USA), anti-tERK (Cat#4695, CST, Boston, MA, USA), anti-GPR37 (Cat#14820-1-AP, Proteintech, Wuhan, China), anti-GAPDH (Cat# 10494-1-AP, Proteintech, Wuhan, China). After 1 h of incubation at room temperature, with a secondary antibody conjugated with horseradish peroxidase, the protein-blotting signals were viewed by the Tanon-5200Multi chemiluminescent imaging system (Shanghai, China) in the presence of ECL substrate (Cat# 32132, Thermo Fisher, USA).

### 2.9. cAMP Production Assays

For cAMP quantification, macrophages were seeded in poly-l-lysine-coated wells of 96-well plates at a cell density of 1 × 10^4^/well. By the treatment of OCN (10 nM) for 30 min in the presence of forskolin, the change of intracellular cAMP concentration was measured using the cAMP-Glo Max Assay Kit (Promega, Cat# V1682) following the manufacturer’s instructions. All the experiments were repeated at least four times in triplicate, and data were analyzed using Graphpad Prism Software.

### 2.10. Macrophage Phagocytosis Assay

The phagocytosis assay was performed as described in previous studies [61,62]. In brief, the pHrodo^TM^ Red Zymosan Bioparticles^TM^, with a diameter of 3 μm (Thermos scientific, Cat# P35364) was washed and reconstituted in RPMI medium. Particles were centrifuged at 1000× *g* for 1 min onto adherent peritoneal macrophages, at a ratio of 10:1, to synchronize binding and internalization. After 30 min incubation at 37 °C, non-adherent particles or cells were removed by washing with cold PBS, and cells were then fixed with 2% PFA. The particles ingested by macrophages were observed under a bright area via a light Zeiss fluorescence microscope system (Axio observer 3), with at least 10 fields (20×) in each sample.

### 2.11. Statistical Analysis

Statistical analyses were carried out using the SPSS version 13.0 software 261 package (SPSS, Chicago, IL, USA) for Windows. All data are indicated as mean ± SD unless otherwise stated. The number of each sample per group is presented in the figure legends. When only two groups were compared, the statistical differences were analyzed with the double-sided Student’s *t*-test. Comparisons among multiple groups were assessed using one-way ANOVA with Tukey’s post hoc test. For all experiments, *p* value < 0.05 was considered a significant difference.

## 3. Results

### 3.1. OCN Attenuates LPS-Induced Acute Inflammation in Both WT and OCN^−/−^ Mice

**Figure 1:**
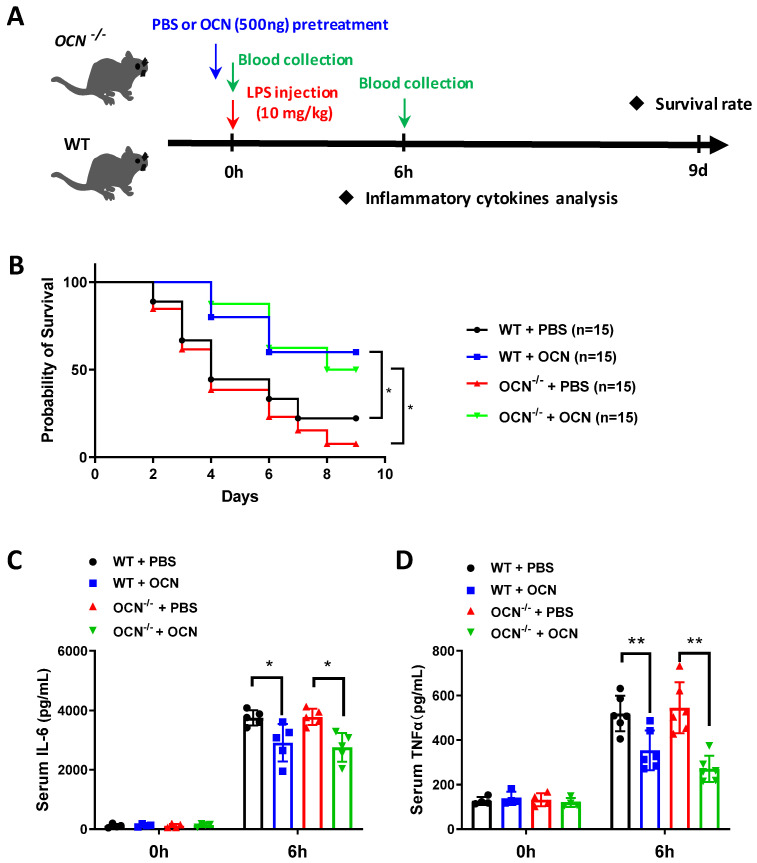
OCN attenuates LPS-caused detrimental effects in WT and *OCN*^−/−^ mice. (**A**) Experimental design for the LPS-induced acute inflammation. The *OCN*^−/−^ and WT mice were injected intraperitoneally with PBS or OCN (500 ng) followed by LPS (10 mg/kg) treatment. Mice survival rate and serum inflammatory were analyzed in each time point, accordingly. (**B**) Survival curves of *OCN*^−/−^ and WT mice treated with PBS or OCN in response to LPS stimulation. Sample sizes are presented in brackets (*n* = 15). * *p* < 0.05, as compared to PBS. (**C**,**D**) ELISA analysis of serum levels of IL-6 (**C**) and TNFα (**D**) in *OCN*^−/−^ and WT mice with PBS or OCN pretreatment after 6h of LPS administration. Sample sizes are indicated as dots in each column. Data are presented as mean ± SD. * *p* < 0.05 and ** *p* < 0.01, as compared to PBS.

To investigate the role of OCN in inflammation, adult *OCN*^−/−^ and WT mice were used for experimental studies. The deletion of the *OCN* gene in *OCN*^−/−^ mice has been validated in our previous work [24]. Firstly, we examined whether the *OCN* gene deletion had an influence on immune cells by performing flow cytometry analysis. The results showed that the number of macrophages was constantly increased in lymph, spleen, and blood of *OCN*^−/−^ mice, as compared with WT (Supplementary Figure S1A–C). Then, we wondered how the *OCN*^−/−^ mice would respond to acute inflammation induced by LPS stimulation (Figure 1A). As shown in Figure 1B, similar mortality (up to 90%) was observed between the *OCN*^−/−^ and WT mice, after the intraperitoneal (I.P.) injection of LPS (10 mg/kg), for 9 days. However, these high mortalities were significantly decreased by pretreatment of OCN (500 ng, Figure 1B). In addition, the level of serum inflammatory cytokines were also analyzed after 6h of LPS stimulation. No significant difference was found in the production of serum IL-6 and TNFα between *OCN*^−/−^ and WT mice before LPS injection (Figure 1C,D). However, LPS treatment caused a remarkable increase in IL-6 and TNFα in both *OCN*^−/−^ and WT mice, which, in turn, were significantly decreased by the infusion of OCN (Figure 1C,D). Thus, these data indicate that OCN plays an inhibitory role in the regulation of LPS-caused high mortality and the production of pro-inflammatory factors.

### 3.2. OCN Inhibits the Release of Pro-Inflammatory Factors in LPS-Treated Macrophages

**Figure 2:**
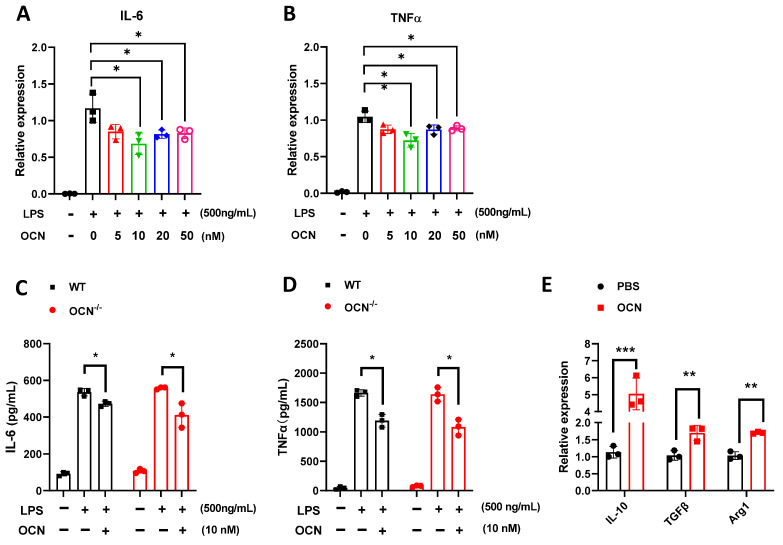
OCN inhibits the production of pro-inflammatory factor in macrophage. (**A**,**B**) Effects of OCN treatment on the mRNA expression of pro-inflammatory genes, i.e., IL-6 (**A**) and TNFα (**B**), in LPS (500 ng/mL) treated peritoneal macrophages from WT mice. Data are presented as means ± SD (*n* = 3 biological replicates). * *p* < 0.05 and ** *p* < 0.01, as compared to PBS. (**C**,**D**) Cytokine levels in a culture medium of peritoneal macrophage from WT and *OCN*^−/−^ mice. Peritoneal macrophages were treated with LPS (500 ng/mL), together PBS or OCN (10 nM) for 12 h, and the levels of IL-6 (**C**) and TNFα (**D**) in culture medium were measured by ELISA analysis. Data are presented as means ± SD. (*n* = 3 biological replicates) * *p* < 0.05, as compared to PBS. (**E**) The mRNA expression analysis of genes associated with anti-inflammatory in peritoneal macrophages isolated from WT mice. Data are presented as means ± SD (*n* = 3 biological replicates). ** *p* < 0.01 and *** *p* < 0.001, as compared to PBS.

Macrophages have been proven to play a crucial role in regulating inflammation reaction in responding to various stress conditions [67,68,69], and we thus assumed that macrophage might mediate the anti-inflammatory actions of OCN. As expected, we found that LPS stimulation (500 ng/mL) dramatically increased the mRNA expression of IL-6 and TNF-α in peritoneal macrophages isolated from WT mice (Figure 2A,B), and in RAW264.7 cells (Supplementary Figure S2), while these upregulations were downregulated by OCN treatment (Figure 2A,B and Figure S2). Based on these findings, OCN at a concentration of 10 nM was used in the following cell experiments unless otherwise stated. Moreover, we further observed that OCN treatment significantly inhibited LPS-induced secretion of IL-6 and TNF-α in a culture medium of peritoneal macrophages from WT mice and *OCN*^−/−^ (Figure 2C,D). By contrast, the anti-inflammatory related genes, i.e., IL-10, transforming growth factor (TGF) β, and arginase (Arg1), were significantly upregulated by OCN treatment (Figure 2E). Together, these results suggest that OCN could act on macrophages to inhibit the pro-inflammatory cytokine production in response to LPS stimulation.

### 3.3. GPR37 Mediates OCN’s Signal in Macrophages

**Figure 3:**
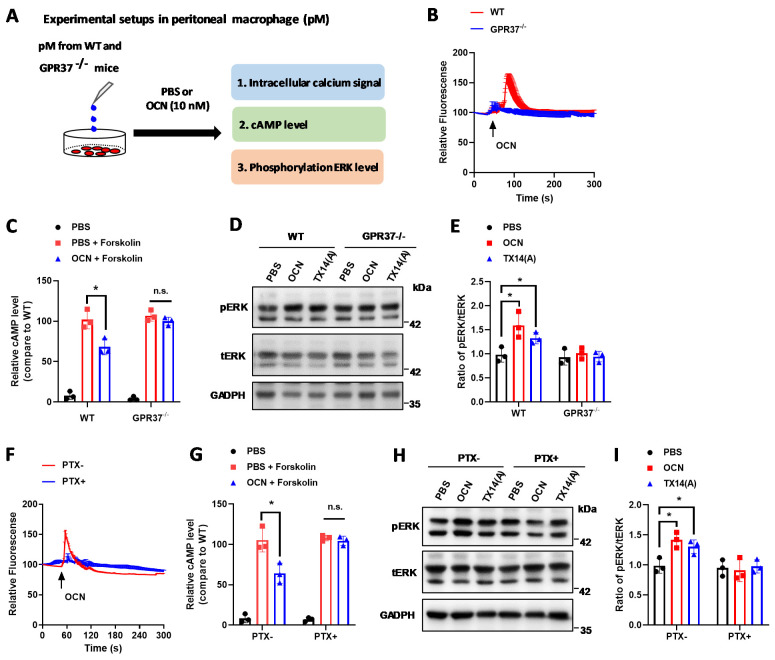
GPR37 mediates OCN-induced intracellular responses in macrophages. (**A**) Overview of the experimental design testing intracellular signals in macrophages. Peritoneal macrophages were isolated from WT and *GPR37*^−/−^ mice to study the changes of intracellular calcium level (iCa^2+^), cAMP production, and pERK level in response to OCN treatment. (**B**) Representative response curves of OCN-triggered iCa^2+^ changes in macrophages from WT and *GPR37*^−/−^ mice. (**C**) OCN-triggered inhibition of cAMP production in macrophages from WT and *GPR37*^−/−^ mice. Data are presented as mean ± SD (*n* = 3 biological replicates). * *p* < 0.05, as compared to PBS, and n.s. indicates not significant. (**D**,**E**) Representative immunoblot images (**D**) and relative quantification (**E**) showing that OCN-triggered an increase in the pERK level in macrophages from WT but not in that from *GPR37*^−/−^ mice. Data are presented as mean ± SD (*n* = 3 biological replicates). * *p* < 0.05, as compared to PBS. (**F**) Representative response curves of OCN-triggered iCa^2+^ changes in PTX pretreated macrophage from WT mice. (**G**) OCN-triggered inhibition of cAMP production in PTX pretreated macrophage from WT mice. Data are presented as mean ± SD (*n* = 3 biological replicates). * *p* < 0.05, as compared to PBS, and n.s. indicates not significant. (**H**,**I**) Representative immunoblot images (**H**) and relative quantification (**I**) showing that PTX pretreatment blocked the OCN-triggered increase in the pERK level in macrophages from WT mice. Data are presented as mean ± SD (*n* = 3 biological replicates). * *p* < 0.05, as compared to PBS.

Next, we asked what receptor transduces the signal of OCN in macrophages. As our previous finding showed that OCN can trigger intracellular signaling through binding with GPR37 [24], while the GPR37 has been reported to be involved in macrophage-associated functions [62]. Thus, we investigated if OCN’s signaling, including the change of intracellular calcium (iCa^2+^), cAMP, and phosphorated ERK (pERK) levels, could be mediated by GPR37 in macrophages (Figure 3A). The GPR37 was first confirmed to be expressed in macrophages from WT but not in that from *GPR37*^−/−^ mice (Supplementary Figure S3). By the transfection of GCamMP6s, an ultrasensitive fluorescent calcium sensor [70], we found that OCN stimulation triggered a fast and robust increase in iCa^2+^ in macrophages from WT but not in that from *GPR37*^−/−^ mice (Figure 3B). Moreover, OCN treatment significantly inhibited forskolin-induced cAMP production and increased pERK level in macrophages from WT mice, and the prosaptide TX14(A), a reported ligand for GPR37 [58], was parallelly used as a positive control (Figure 3C–E). However, these actions of OCN were absent in macrophages derived from *GPR37*^−/−^ mice (Figure 3C–E). Since GPR37 is coupled with the G_i/o_ signaling pathway [24,58], we further observed that the pretreatment with pertussis toxin (PTX), an inhibitor of the G_i/o_ pathway, abolished all these OCN elicited changes of intracellular signals in macrophages (Figure 3F–I). Thus, these findings suggested that GPR37 mediates calcium and cAMP signaling of OCN in macrophages.

**Figure 4:**
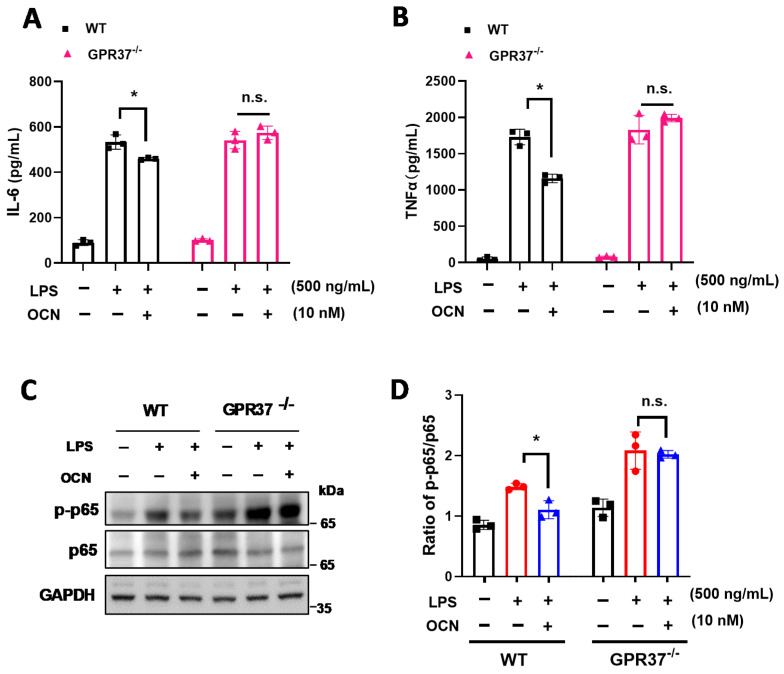
GPR37 mediates the inhibitory effects of OCN on pro-inflammatory factors in macrophage. (**A**,**B**) Cytokine levels in the culture medium of peritoneal macrophages from WT and *GPR37*^−/−^ mice. Cells were treated with LPS (500 ng/mL, 37 °C, 24 h) together with PBS or OCN (10 nM). ELISA analysis was performed to measure the level of IL-6 (**A**) and TNF-α (**B**). Data are presented as mean ± SD (*n* = 3 biological replicates), * *p* < 0.05, as compared to PBS, and n.s. indicates not significant. (**C**,**D**) Representative immunoblot images (**C**) and quantitative analysis (**D**) of NFκB p65 level in macrophages treated with LPS in the presence of OCN. Data are presented as mean ± SD (*n* = 3 biological replicates), * *p* < 0.05 as compared to PBS, and n.s. indicates not significant.

To further determine whether GPR37 is necessary for OCN’s functions, we treated WT and *GPR37*^−/−^ mice-derived peritoneal macrophages with LPS, in the presence of OCN (10 nM), for pro-inflammatory cytokines measurement. We found that OCN treatment significantly inhibited LPS-induced secretion of IL-6 and TNFα in culture medium of macrophage from WT, while this observation was absent in macrophages from *GPR37*^−/−^ mice (Figure 4A,B). Considering that NFκB is a well-known downstream transcriptional regulator of inflammatory signaling [71,72], we further examined whether NFκB signaling was involved in OCN signals. The results showed that the LPS-stimulated p-p65 level was significantly inhibited by OCN in macrophages from WT but not in that from *GPR37*^−/−^ mice (Figure 4C,D). Thus, these results indicated that GPR37 could mediate the anti-inflammatory effect of OCN in macrophages, probably through the regulation of NFκB signals.

### 3.4. GRP37 Is Required for OCN’s Function in Macrophages

**Figure 5:**
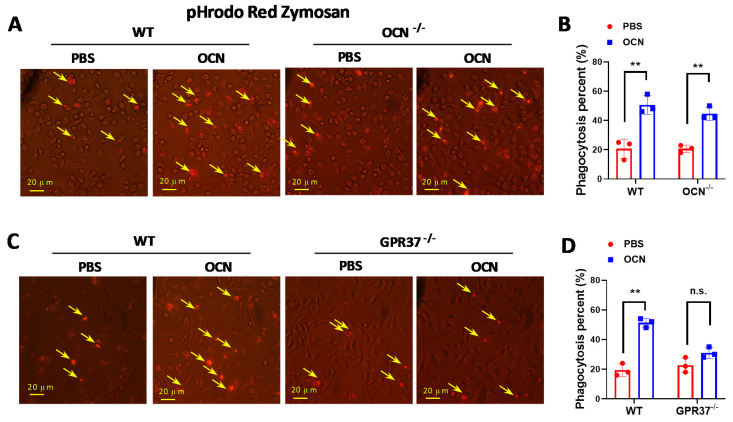
OCN promotes macrophage phagocytosis via GPR37. (**A**) Representative images of in vitro phagocytosis assay, in which pHrodo zymosan particles were incubated with peritoneal macrophages from WT or *OCN*^−/−^ mice, treated with PBS or OCN (10 nM, 30 min, 37 °C). Red fluorescence suggests an intracellular update indicating phagocytosis. Scale bars: 20 μm. (**B**) Quantification of macrophage phagocytic activity of zymosan positives cells. Data are presented as mean ± SD (*n* = 3 biological replicates), ** *p* < 0.01, as compared to PBS. (**C**) Representative images of an in vitro phagocytosis assay, in which pHrodo zymosan particles were incubated with peritoneal macrophages from WT or *GPR37*^−/−^ mice, treated with PBS or OCN (10 nM, 30 min, 37 °C). Scale bars: 20 μm. (**D**) Quantification of macrophage phagocytic activity of zymosan positives cells. Data are presented as mean ± SD (*n* = 3 biological replicates), ** *p* < 0.01, as compared to PBS, and n.s. indicates not significant.

Previous studies have shown that the activation of GPR37 by its ligand/agonist can augment macrophage phagocytosis [61,62]. Thus, we speculated OCN should exert similar effects through GPR37 in macrophages. As was our expectation, we observed that OCN treatment (10 nM for 30 min) increased the uptake of fluorescently-labeled zymosan particles in macrophages from both WT and *OCN*^−/−^ mice (Figure 5A,B), while this effect was dampened in macrophages from *GPR37*^−/−^ mice (Figure 5C,D). 

### 3.5. GPR37 Is Required for OCN’s Protective Role against LPS-Induced Acute Inflammation In Vivo

**Figure 6:**
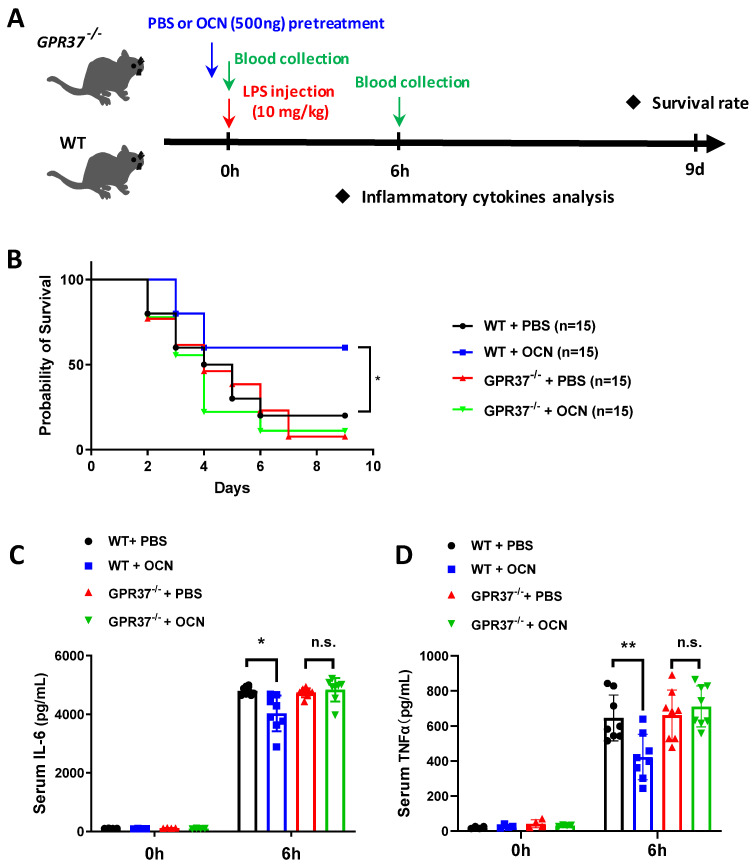
The protective function of OCN against LPS is absent in *GPR37*^−/−^ mice. (**A**) Experimental design for LPS-induced acute inflammation in *GPR37*^−/−^ and WT mice. After intraperitoneal injection of PBS or OCN (500 ng), followed by LPS (10 mg/kg) treatment, mice survival rate and serum inflammatory cytokine level were analyzed accordingly. (**B**) Survival curves of *GPR37*^−/−^ and WT mice treated with PBS or OCN in response to an LPS challenge. Sample sizes are presented in brackets (*n* = 15). * *p* < 0.05, as compared to PBS. (**C**,**D**) After 6h of LPS injection, with or without OCN pretreatment, serum IL-6 (**C**) and TNFα (**D**) levels were analyzed in *GPR37*^−/−^ and WT mice. Sample sizes are indicated as dots in columns. * *p* < 0.05 and ** *p* < 0.01, as compared to PBS; n.s. indicates not significant.

The above data showed that OCN, via GPR37, could regulate macrophage functions in vitro, so we next sought to examine whether GPR37 also mediated OCN’s function in vivo. The WT and *GPR37*^−/−^ mice were pretreated with PBS or OCN (500 ng), and then, they were exposed to the LPS challenge for the analysis of survival and inflammatory cytokines release (Figure 6A). Compared with OCN treated *GPR37*^−/−^ mice, OCN-treated WT mice had a significantly improved survival rate (Figure 6B). Moreover, we found that OCN protected against LPS-induced production of IL-6 and TNFα levels in WT mice, while these beneficial effects of OCN were abolished in *GPR37*^−/−^ mice (Figure 6C,D). Therefore, these data indicated that GPR37 mediated the anti-inflammatory function of OCN in LPS-challenged mice.

### 3.6. Adoptive Transfer of OCN-Treated Macrophages Alleviates LPS-Induced Acute Inflammation

**Figure 7:**
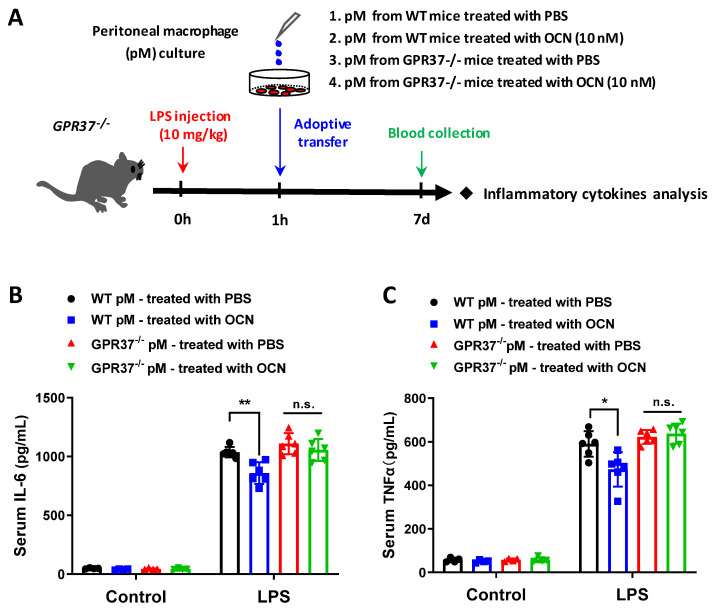
Adoptive transfer of OCN-treated macrophages confers protection against LPS stimulation. (**A**) Experimental design, to test whether adoptive transfer of macrophages pretreated with OCN, could attenuate LPS-induced acute inflammation. Peritoneal macrophages were isolated from WT or *GPR37*^−/−^ mice and then treated with PBS or OCN (10 nM) for 24 h, followed by a washout of OCN and an adoptive transfer of macrophages (I.P.) into *GPR37*^−/−^ mice challenged with LPS for 1 h. (**B**,**C**) ELISA analysis of serum IL-6 (**B**) and TNFα (**C**) levels, in *GPR37*^−/−^ mice with adoptive transfer, of macrophages derived from WT or *GPR37*^−/−^ mice. Sample sizes are indicated as dots in each column. Data are presented as mean ± SD. * *p* < 0.05 and ** *p* < 0.01, as compared to PBS; n.s. indicates not significant.

To further confirm whether OCN/GPR37 axis in macrophages confers protection against LPS stimulation, we carried out an adoptive transfer experiment by I.P. injection of peritoneal macrophages (2 × 10^5^) into *GPR37*^−/−^ mice after 1 h of LPS inoculation (Figure 7A). The cells were isolated from WT or *GPR37*^−/−^ mice, and treated with OCN (10 nM) or PBS, for 24 h before transplantation. Blood was collected for inflammatory cytokine analysis, after cell transplantation, for 6 h (Figure 7A). We observed that an LPS-caused increase in IL6 and TNFα levels was significantly inhibited by the adoptive transfer of OCN-treated—but not PBS-treated—macrophages from WT mice (Figure 7B,C). By contrast, the adoptive transfer of macrophages from *GPR37*^−/−^ mice, either by OCN or by PBS treatment, have no protective effects against LPS stimulation (Figure 7B,C). Together, these results demonstrate that the regulatory axis of OCN/GPR37 in macrophages is important for relieving LPS-induced acute inflammatory responses.

## 4. Discussion

**Figure 8:**
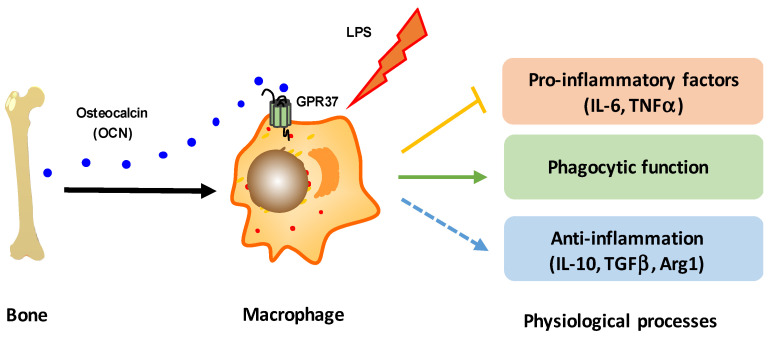
A schematic diagram illustrating that the bone-derived hormone OCN, via the activation of GPR37 in macrophages, plays a protective function against LPS challenge through the regulation of macrophage inflammatory reactions and phagocytic function.

Besides the structural function as a bone matrix, OCN has been increasingly recognized as a versatile functional hormone in numerous cellular activities and biological processes [3,4,5,6,7]. Although great advances have been achieved in deciphering the function of OCN in various peripheral organs [10,14,19,20,22,23,73] and in the CNS [6,24,26], the exact role for OCN in the regulation of inflammation is still under debate. In this study, we provide evidences that OCN, indeed, plays an anti-inflammatory role in LPS-induced acute inflammation. Our results demonstrate that the bone-derived hormone OCN can interact with GPR37 to activate intracellular downstream signal transduction in macrophages, thus modulating inflammation reactions and phagocytic function in response to LPS stimulation (Figure 8). Moreover, this study also implied that the interaction between the bone-secreted OCN and macrophage-expressed GPR37 could link the skeletal endocrine function and immunological activity, thereby providing a distinctive viewpoint for the understanding of mutual communication between bone biology and the immune system.

In the present work, we found that I.P. injection of OCN decreased the mortality in both WT and *OCN*^−/−^ mice challenged with a lethal dose of LPS (Figure 1), suggesting a protective role for OCN against LPS-induced death. Moreover, we further observed that OCN treatment significantly inhibited the expression and secretion of IL-6 and TNFα levels in LPS-stimulated mice and macrophages (Figure 1 and Figure 2), indicating a negative relation between OCN and pro-inflammatory cytokines release. These findings are consistent with a number of previous studies. For instance, some clinical observations have shown that OCN is inversely correlated with systemic inflammatory markers (e.g., IL-6 and CRP) in patients with diabetes, obesity, or metabolic syndrome [30,31,32,33,34,35,36], while OCN treatment could substantially downregulate inflammatory related genes and transcription factors in an obese mice model [37] and in TNFα-stimulated primary cultured adipocytes [37,38]. However, it should be noted that certain studies argue that OCN is non-functional in inflammation or even positively correlated with the development of inflammatory processes. As a recent study by Millar et al. reported that OCN has no influence on acute or chronic inflammatory human vascular cells as induced by interferon-γ and TNFα [40]. Furthermore, it was showed that OCN level is increased during the calcification of atherosclerotic plaques, which is positively correlated with the activation of inflammatory processes and the release of pro-inflammatory biomarkers [39]. The discrepancies among these studies could be due to the difference in experimental designs and inflammatory models, resulting in the differential performance of OCN. Interestingly, we showed that mice, with the deletion of the OCN gene, has no more of a deteriorative inflammatory response than WT mice under LPS expose (Figure 1 and Figure 2), which might be attributed to the increased number of macrophages that compensated the immune activity in *OCN*^−/−^ mice (Supplementary Figure S1), yet the exact mechanism should be investigated in a future study.

The phenotypes and functions of macrophages can be rapidly regulated by surrounding micro-environmental stimuli [74]. In this work, we showed that OCN treatment of macrophages resulted in the downregulation of pro-inflammatory cytokines but the upregulation of anti-inflammatory related genes (Figure 2E), indicating a potential role of OCN in promoting a phenotype of alternatively activated macrophages. Similar effects of OCN have also been previously reported in whole organ adipose tissue [38]. Moreover, we also found macrophage phagocytic function was increased by OCN stimulation (Figure 5). Importantly, our subsequent adoptive transfer experiments further revealed that the infusion of OCN-treated macrophages could attenuate the inflammatory responses in LPS-exposed mice (Figure 7). Thus, it was suggested that the protective role of OCN against LPS could be, at least partially, attributed to its regulation of macrophage phenotype and function. Nevertheless, the physiological importance of OCN in regulation of macrophage phenotype and phagocytic function, and the underlying molecular mechanisms, ought to be explored with expanding experiments.

Recently, two research studies point out that macrophage-expressed GPR37 plays a crucial role in regulating inflammation [61,62]. These works reported that the activation of GPR37, by its ligand NPD1 or agonist ARU, can protect against bacterial infections, sepsis, and inflammatory pain-like behaviors [61,62]. Interestingly, our previous work identified that GPR37 is a specific receptor for OCN in oligodendrocyte, and it mediates the function of OCN in the central nervous system [24]. By using the HEK293 cell-based heterologous expressing system, we provided robust evidence that OCN can interact with GPR37 to elicit various intracellular downstream signaling [24]. Thus, it is reasonable to speculate that OCN may also take actions in macrophages via GPR37. As supporting evidence, our work showed that OCN treatment could trigger rapid change of iCa^2+^, cAMP, and pERK levels in peritoneal macrophage from WT but not in that from *GPR37*^−/−^ mice (Figure 3); the protective effects of OCN against LPS stimulation were absent in *GPR37*^−/−^ mice (Figure 4, Figure 5 and Figure 6). Thus, these findings suggested GPR37, indeed, transduced OCN’s signal and its anti-inflammatory functions in macrophages. In vivo study further demonstrated that the adoptive transfer of OCN-treated macrophage from WT mice, rather than *GPR37*^−/−^ mice, could also attenuate LPS-induced adverse effects (Figure 7). It is worth noting that OCN treatment could suppress LPS-induced upregulation of p-p65 level in macrophages from WT but not in that from *GPR37*^−/−^ mice (Figure 4), indicating p-p65 could be a key regulator in the downstream of the OCN/GPR37 axis. Nevertheless, more in-depth work should be conducted to elucidate the underlying molecular regulatory network of how p65 is activated by the interaction between OCN and GPR37. 

The current work has a number of limitations. First of all, the anti-inflammatory effects of OCN were observed by OCN pretreatment before the occurrence of inflammation, either LPS stimulation in mice or peritoneal macrophages. These observations hint at a preventive and protective effect of OCN on the inflammation activation. However, further therapeutic studies should be performed to examine whether OCN supplementation could be able to delay or reverse the progress of inflammation in a developed, and even advanced, inflammatory state. Furthermore, the functional role of OCN, related to inflammation activation and the underlying regulatory mechanisms, can be further explored in a future study. Secondarily, although the LPS-induced acute inflammation is a convenient and common method to mimic inflammatory diseases such as sepsis, the LPS-based model may only partially reflect the development of pathophysiological features in human inflammation, of which the progress of inflammation could be either an acute or chronic procedure. Hence, the finding that OCN treatment could attenuate LPS-induced acute inflammation, through activating GPR37 in macrophages, ought to be further validated in other chronic inflammatory animal models or clinical studies. Finally, additional experiments could be carried out to put insights into this study, i.e., (i) experiments for the investigation of whether OCN, indeed, plays a role in the regulation of macrophage polarization (i.e., M1/M2 phenotype), as well as (ii) experiments for the investigation of how OCN facilitates macrophage phagocytic functions (e.g., cargo influx and efflux-related genes in macrophages).

## 5. Conclusions

In conclusion, our study provides evidence that OCN is essentially involved in the regulation of LPS-induced acute inflammation. Moreover, our results demonstrate that the beneficial effects of OCN on inflammatory responses can be attributed to its activation of GRP37 in macrophages. This study also suggests that activation of the OCN/GPR37 axis could be a potential strategy to serve as a therapeutic intervention for inflammatory disease, although further validation in animal models and clinical studies should be performed instantly.

## Figures and Tables

**Table 1:** Primer sequences used in this study.

Gene Primer Name	Sequence (5′–3′)
*TNFa-F*	GCCTCTTCTCATTCCTGCTT
*TNFa-R*	TGGGAACTTCTCATCCCTTTG
*IL-6-F*	CAAAGCCAGAGTCCTTCAGAG
*IL-6-R*	GTCCTTAGCCACTCCTTCTG
*Arg1F*	AAGAATGGAAGAGTCAGTGTGG
*Arg1R*	GGGAGTGTTGATGTCAGTGTG
*IL10-F*	AGGCGCTGTCATCGATTT
*IL10-R*	CACCTTGGTCTTGGAGCTTAT
*TGFβ-F*	CCTGAGTGGCTGTCTTTTGA
*TGFβ-R*	CGTGGAGTTTGTTATCTTTGCTG
*GAPDH-F*	AACAGCAACTCCCACTCTTC
*GAPDH-R*	CCTGTTGCTGTAGCCGTATT

## Data Availability

All data needed to evaluate the conclusions in the paper are present in the paper and/or the Supplementary Materials.

## Supplementary Materials

The following supporting information can be downloaded at: https://www.mdpi.com/article/10.3390/biomedicines10051006/s1, Figure S1: Flow cytometry analysis of dendritic cell, neutrophils and macrophage in different tissues, Figure S2: OCN inhibits the mRNA expression of pro-inflammatory genes in RAW264.7 cells, Figure S3: The expression of GPR37 in macrophages were isolated from WT and *GPR37*^−/−^ mice.

## Abbreviations

WT	wild type
I.P.	Intraperitoneal
OCN	osteocalcin
ARU	agonist artesunate
IL-6	interleukin-6
PTX	pertussis toxin
CRP	C-reactive protein
LPS	lipopolysaccharide
TNFα	tumor necrosis factor
CNS	central nervous system
GPR	G protein coupled receptor
TGFβ	transforming growth factor
cAMP	cyclic Adenosine monophosphate
ELISA	enzyme linked immunosorbent assay
qPCR	real-time quantitative polymerase chain reaction
SDS-PAGE	sodium dodecyl sulphate-polyacrylamide gel electrophoresis
