# Supplementary material for: Osteocalcin Alleviates Lipopolysaccharide-Induced Acute Inflammation via Activation of GPR37 in Macrophages
Source: Biomedicines. 2022 Apr 27;10(5):1006. doi: 10.3390/biomedicines10051006 (PMC9138386; doi:10.3390/biomedicines10051006)
Supplement: Supplementary file 1 [file biomedicines-10-01006-s001.zip › biomedicines-1673993-supplementary.pdf]

Supplementary Materials of this study

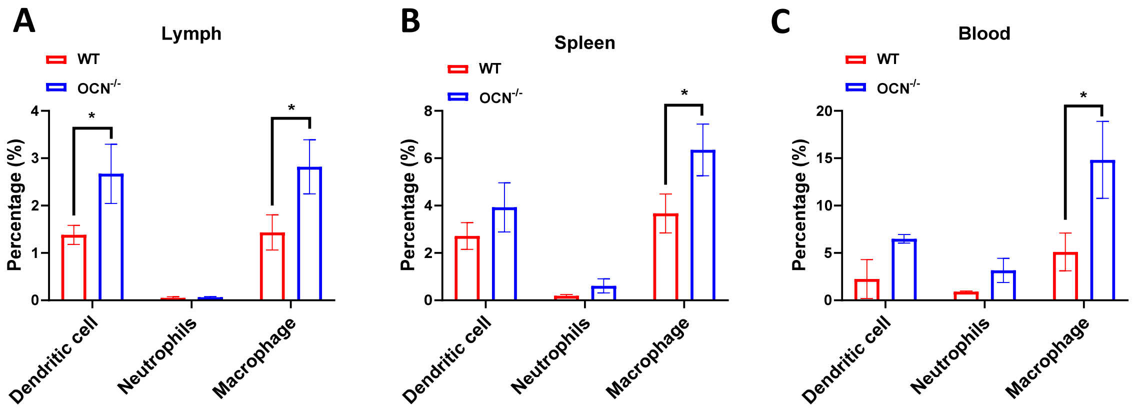

Supplementary Figure S1. Flow cytometry analysis of dendritic cell, neutrophils and macrophage in lymph (A), spleen (B) and blood (C) from WT and *OCN*$^{-/-}$ mice. n = 2 mice; Values represent mean ± SD. * p<0.05 as compared to WT.

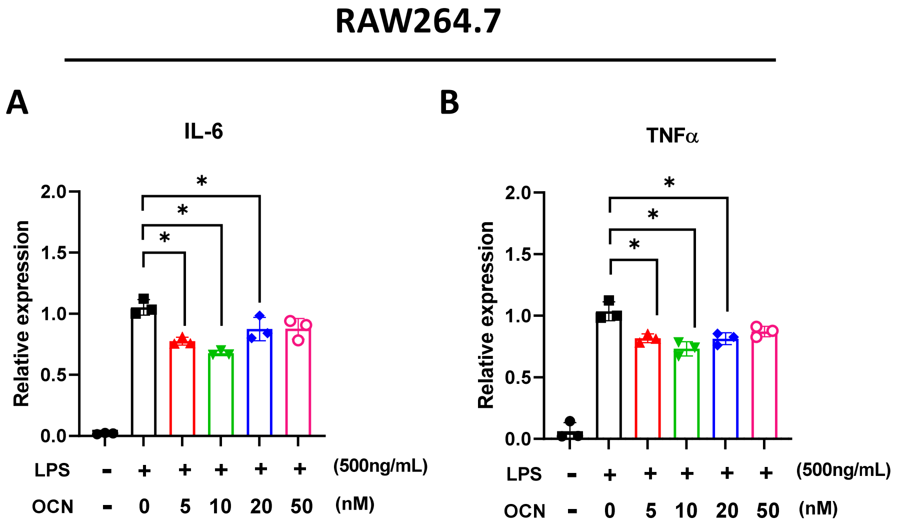

Supplementary Figure S2. OCN inhibits the mRNA expression of pro-inflammatory genes, i.e., IL-6 (**A**) and TNF (**B**) in LPS (500 ng/mL) treated RAW264.7 cells. Data are presented as means ± SD (n = 3 biological replicates). * p<0.05 as compared to PBS.

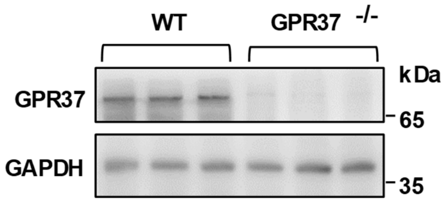

Supplementary Figure S3. Peritoneal macrophages were isolated from WT and GPR37-/- mice and then western blot was performed to confirm the expression of GPR37 in macrophage (n = 3 mice).
